# Low-dose gadobenate dimeglumine-enhanced MRI of the kidney for the differential diagnosis of localized renal lesions

**DOI:** 10.1007/s11547-015-0548-7

**Published:** 2015-06-19

**Authors:** Guenther Schneider, Thorsten Probst, Miles A. Kirchin, Jonas Stroeder, Peter Fries, Arno Buecker

**Affiliations:** Department of Diagnostic and Interventional Radiology, Saarland University Medical Center, 66421 Homburg/Saar, Germany; Global Medical and Regulatory Affairs, Bracco Imaging SpA, Milan, Italy

**Keywords:** Gadobenate dimeglumine, Kidney, Renal MRI, Renal cell carcinoma

## Abstract

**Objective:**

To evaluate low-dose gadobenate dimeglumine-enhanced MRI for the differential diagnosis of malignant renal tumors.

**Methods:**

Sixty-two consecutive patients with unclear diagnosis at MDCT/ultrasound underwent dynamic CE-MRI of the kidneys with 0.05 mmol/kg gadobenate dimeglumine. Retrospective image evaluation was performed by two blinded readers. Lesion diagnosis at CE-MRI was correlated with findings from histology following tumor resection or from imaging follow-up after at least 1 year. Assessments were performed of diagnostic quality and level of diagnostic information.

**Results:**

Thirty-nine (63 %) patients were correctly diagnosed with malignant lesions (36 with RCC, 2 with renal metastases, 1 with lymphoma) while 14 (22.6 %) patients were correctly diagnosed with benign (*n* = 12) or no (*n* = 2) lesions. Eight patients were considered false positive (5 with oncocytoma, 3 with atypical AML) and 1 patient false negative (atypical RCC). The sensitivity, specificity, accuracy, PPV, and NPV for the diagnosis of malignant renal lesions were 97.5 % (39/40), 63.6 % (14/22), 85.5 % (53/62), 83.0 % (39/47), and 93.3 % (14/15), respectively. Images were excellent in 60 and good in 2 patients. Minimal artifacts that did not compromise diagnosis were noted in 4/62 patients.

**Conclusion:**

Low-dose gadobenate dimeglumine-enhanced MRI is effective for the differential diagnosis of malignant renal tumors.

## Introduction

Although renal cell carcinoma (RCC) is the most common malignant epithelial tumor of the kidney accounting for 85–90 % of all solid renal tumors in adults and representing 5 % of all cancers in men and 3 % in women [1, 2], benign solid neoplasms such as angiomyolipoma (AML) and oncocytoma represent 10–14 % of all resected solid renal tumors [3–5]. A major clinical need therefore is to accurately differentiate RCC and other malignant renal lesions which typically require urgent surgical attention from benign solid neoplasms for which conservative management is usually indicated.

Whereas contrast-enhanced ultrasound (CEUS; [6–8]) and multi-detector-computed tomography (MDCT; [9–11]) are frequently first-line imaging techniques that reveal the presence of renal masses, often incidentally, subsequent work-up of patients with suspected malignant renal disease is usually performed using contrast-enhanced MR imaging (CE-MRI) because of the wide versatility of this technique and its ability to accurately identify fat within renal tumors. Several studies have demonstrated the effectiveness of CE-MRI not only for the differential diagnosis of renal tumors but also for the accurate differentiation of different RCC sub-types [12–19].

To date, most recent protocols for CE-MRI of the kidney have utilized gadolinium-based contrast agents (GBCAs) at a dose of at least 0.1 mmol/kg bodyweight [13–18]. Moreover, the contrast agents used have invariably been conventional GBCAs such as gadopentetate dimeglumine which have standard r1 relaxivity values of approximately 4.2 L mmol^−1^ s^−1^ at 1.5 T [20]. The aim of our study was to retrospectively determine the diagnostic performance of CE-MRI for the accurate diagnosis of malignant renal masses using a lower dose (0.05 mmol/kg BW) of the much higher relaxivity GBCA gadobenate dimeglumine (MultiHance; Bracco Imaging SpA, Milan, Italy).

## Methods and materials

### Patients

Between January 2008 and December 2013, 184 consecutive patients at our institution underwent abdominal CE-MRI with 0.05 mmol/kg BW gadobenate dimeglumine with specific emphasis on dynamic CE-MRI of the kidneys. Of these 184 patients, 122 (66.3 %) underwent CE-MRI for follow-up of prior surgical resection of known malignant lesions or for routine-scheduled follow-up of known benign lesions. The remaining 62 (33.7 %) patients underwent CE-MRI because of equivocal renal tumors detected at multiphasic MDCT or ultrasound. This retrospective assessment focuses on these 62 patients. Institutional review board approval was obtained for the study. Written informed consent for the use of individual patient imaging data for research purposes was obtained from all patients.

The 62 evaluated patients included 37 men (mean age 58.5 ± 16.1 years; age range 10–79 years) and 25 women (mean age 62.5 ± 11.9 years; age range 37–80 years) with an overall mean age of 60.1 ± 14.8 years (age range 10–80 years).

### MR imaging

MR imaging examinations were performed at 1.5 Tesla (Magnetom Vision, Sonata or Aera; Siemens Medical Systems, Erlangen, Germany). Patients were imaged in the supine position using a surface phased-array coil and the following sequences: (a) coronal T2-weighted half-Fourier single-shot fast spin-echo [repetition time (TR) ms/echo time (TE) ms, 800–1100/60–92; section thickness, 5 mm; gap, 1 mm; matrix, 192 × 256; flip angle, 155°–180°; field of view (FOV), 35–45 cm], (b) axial dual-echo T1-weighted in-phase and opposed-phase gradient-echo (130–205/2.2–2.7, 4.5–5.2; flip angle, 70°; section thickness, 5 mm; gap, 1 mm; matrix, 160 × 256; FOV, 35–45 cm). For contrast-enhanced imaging, a dynamic T1-weighted gradient-echo sequence (160–210/4.5–4.8; flip angle, 70°; slice thickness, 5–6 mm; gap, 1 mm; matrix 154–180 × 256; FOV, 35–45 cm) was used when using a Siemens Magnetom Vision or Sonata. In the equilibrium phase, a T1w gradient-echo sequence with chemically selective fat saturation was acquired (134–160/2.3–2.7; flip angle, 70°; slice thickness, 5 mm; gap, 1 mm; matrix 154–195 × 256–320; FOV, 35–45 cm). With the Magnetom Aera system volume-interpolated breath-hold examinations (VIBE) were performed for both unenhanced and contrast-enhanced imaging. A Dixon T1w sequence with the following parameters was acquired: TR 6.77 ms; TE 2.38 ms; slice thickness 3 mm; FOV, 380 mm^2^; breath-hold acquisition time 21 s, permitting acquisition of the following 4 T1w images at each slice level: in-phase image (standard T1), opposed-phase image, water-only image (fat suppressed T1), fat-only image.

Contrast-enhanced T1-weighted gradient-echo acquisitions were obtained dynamically in the cortico-medullary and nephrographic phases after administration of a bolus of 0.05 mmol/kg bodyweight of gadobenate dimeglumine at a rate of 2 mL/s followed by a 20-mL saline flush. For early examinations, the first pass was timed to the cortico-medullary phase by best guess (25 s post-start of contrast injection) while the nephrographic phase was acquired at approximately 5 min post-contrast injection. With the newer VIBE sequences, multiple phases were acquired and the appropriately timed sequences were chosen from the acquired data.

Routine diffusion-weighted imaging (DWI) for patients with suspected RCC was introduced into our department in September 2010 when the Magnetom Aera was installed. Of the 62 patients included in this assessment, DWI was performed for 29 patients using an echo planar imaging-spin echo (EPI-SE) sequence with free breathing. The parameters for DWI acquisitions were as follows: TR/TE [ms] = 6400/68, section thickness, 6 mm; gap, 0 mm; number of slices, 35; matrix, 192 × 156; averages, 3; FOV [mm × mm], 380 × 308; spatial resolution [mm^3^], 2.0 × 2.0 × 5.0; *b* values [s/mm^2^], 0, 400, 800; bandwidth [Hz/px], 1735; acquisition time [min], 4:30. Parallel imaging (GRAPPA 2) and fat suppression (SPAIR) were used.

### Image evaluation

CE-MRI images were acquired as part of routine clinical practice. Based on CE-MRI diagnosis in conjunction with available findings from prior diagnostic imaging studies, patients were referred either for surgical resection of the detected lesion(s) or for conservative management. Typically, lesions that show no evidence of homogeneous fat distribution on unenhanced MR images but which demonstrate contrast enhancement following contrast administration are malignant in nature. Occasionally, RCC may present with minimal fat in just one area of the lesion. For the purposes of this study, lesions that demonstrated either of these features were considered malignant. Final lesion diagnosis was based on the histopathologic results for the specimen obtained at surgical resection or on imaging follow-up obtained after at least 1 year.

Subsequent retrospective evaluation of images was performed qualitatively by two readers (PF, AB; 8 and 15 years’ experience in abdominal MRI, respectively) in consensus who were not involved in the conduct of the studies and who were fully blinded to the clinical history of the patients, the results of all diagnostic imaging examinations, and to the final clinical diagnosis. Assessments were performed in terms of quality of kidney visualization (insufficient, poor, moderate, good, excellent), presence of artifacts (severe, moderate, minimal, none), extent of diagnostic information (unsatisfactory, partial, satisfactory, complete), and overall diagnostic value (limited, satisfactory, high). Additional assessments were performed of lesion size and of any additional diagnostic information.

### Statistical analysis

Diagnostic performance for the characterization of lesions as malignant or benign was performed at the patient level for all 62 patients using CE-MRI images plus unenhanced axial dual-echo T1-weighted in-phase and opposed-phase gradient-echo images for the analysis of fat content. For this evaluation, patients diagnosed with malignant renal lesions at CE-MRI which were confirmed as malignant at final diagnosis were considered true positive (TP) while patients diagnosed with benign renal lesions or no lesions at CE-MRI which were confirmed as benign or absent at final diagnosis were considered true negative (TN). Patients with renal lesions diagnosed as malignant at CE-MRI which were confirmed as benign at final diagnosis were considered false positive (FP) while patients with renal lesions diagnosed as benign at CE-MRI which were confirmed as malignant at final diagnosis were considered false negative (FN). Based on these findings, determinations were made of the sensitivity, specificity, accuracy, positive predictive value (PPV), and negative predictive value (NPV) of CE-MRI with 0.05 mmol/kg gadobenate dimeglumine for the diagnosis of malignant renal tumors.

## Results

**Fig. 1:**
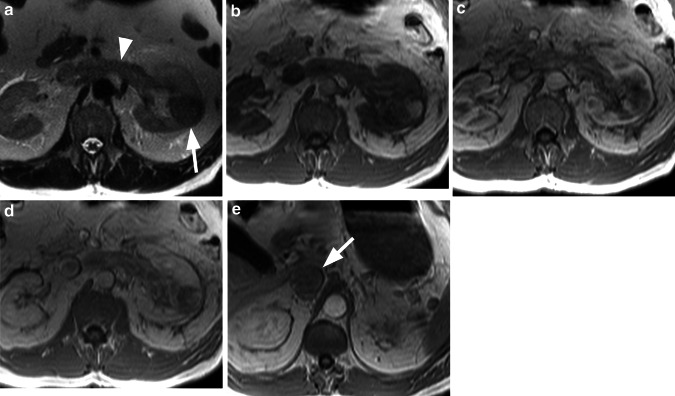
Renal cell carcinoma with a tumor thrombus in the left renal vein and IVC. The T2w HASTE sequence (**a**) reveals a small lesion in the left kidney (*arrow*) together with enlargement of the left renal vein, filled with material that is isointense to the normal renal parenchyma (*arrowhead*). On the corresponding T1w image (**b**) the tumor is hyperintense and no flow void is visible within the left renal vein. After injection of gadobenate dimeglumine at a dose of 0.05 mmol/kg BW the tumor and left renal vein show inhomogeneous enhancement in the early phase (**c**) with early contrast wash-out in the later phase (**d**). This enhancement suggests a RCC with a tumor thrombus in the left renal vein. Note the complete occlusion of the IVC (**e**), with the IVC completely filled with solid tumor material from the tumor thrombus (*arrow* in **e**)

**Fig. 2:**
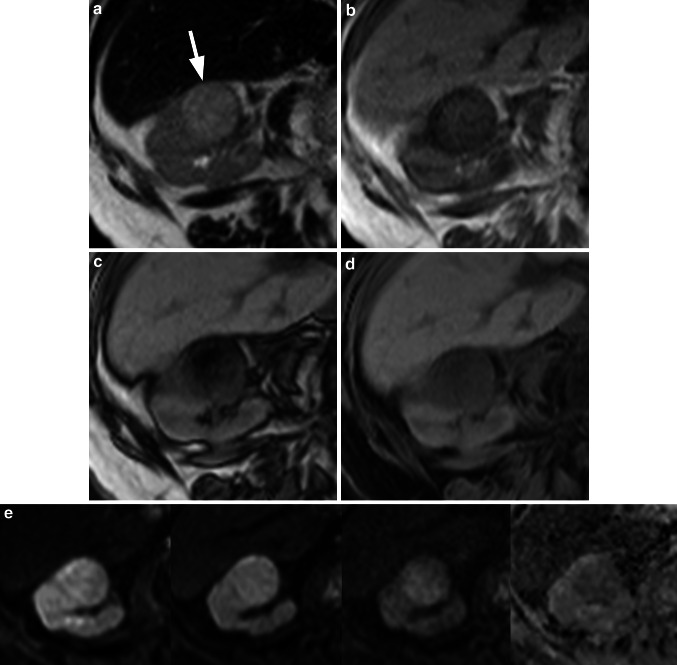

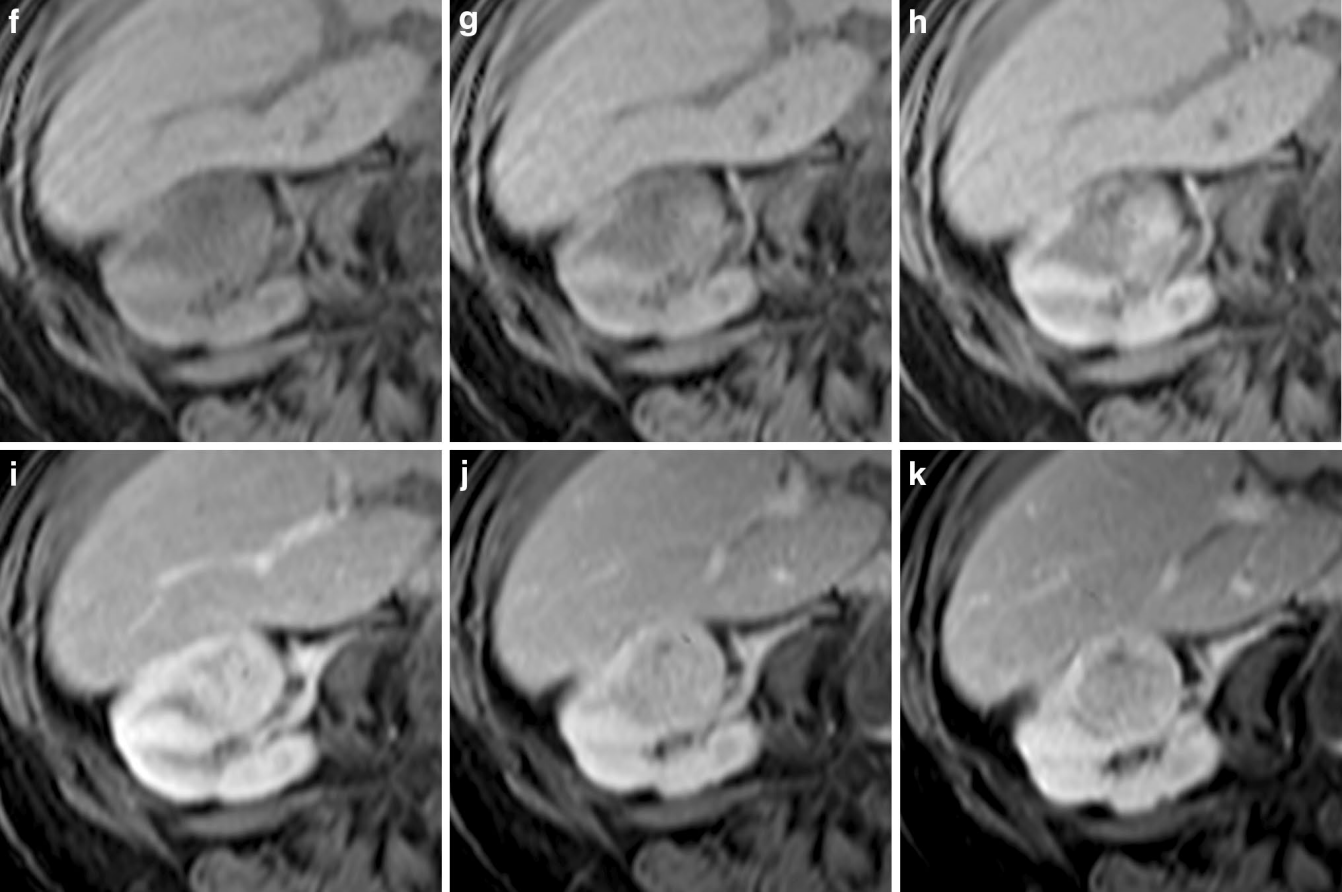
Renal cell carcinoma. On the T2w HASTE sequence (**a**) a hyperintense lesion (*arrow*) is visible in the right kidney. On the VIBE sequence with acquisition of in-phase, opposed-phase, and Dixon fs-images (**b**–**d**) in one single breath-hold, the lesion demonstrates a hypointense rim on the in-phase image (**b**), some hypointense areas in the center on the opposed-phase image (**c**) indicating fat within the tumor, and a homogeneous hypointense signal in the fat suppressed image (**d**). The diffusion-weighted images and the ADC-map (**e**; *b* = 50, 400, 800 +ADC from *left* to *right*) reveal restricted diffusion and a hypointense appearance of the tumor in the ADC-map. Dynamic contrast-enhanced fs VIBE-sequences (**f**–**k**) (0.05 mmol/kg gadobenate dimeglumine) demonstrate tumor hypervascularity with early contrast wash-out. All findings indicate a RCC which was proven on histology after partial nephrectomy

**Fig. 3:**
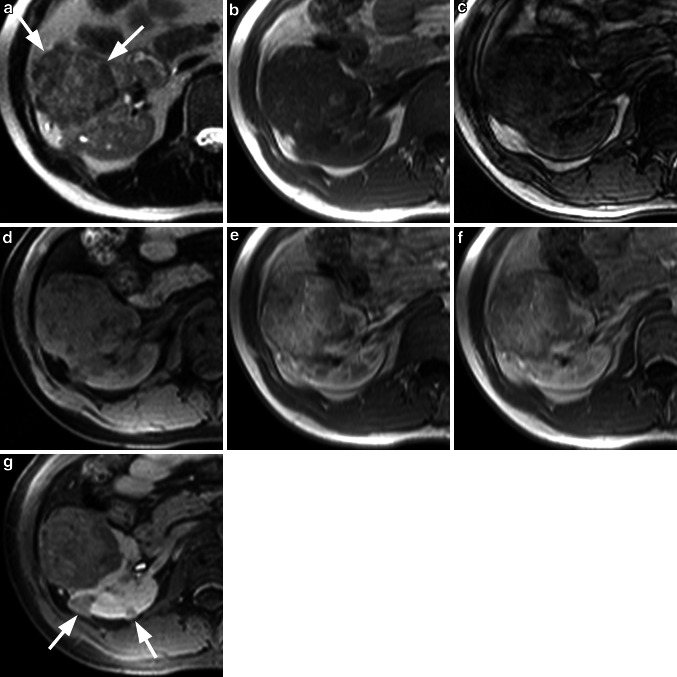
Multiple angiomyolipoma. The T2w HASTE sequence (**a**) shows a large mass (*arrow*) in the right kidney with heterogeneously distributed fat throughout. On the corresponding T1w image (**b**) the tumor shows areas of increased signal intensity, indicating either fat or hemorrhage within the tumor. The areas of increased signal on the T1w image are hypointense on the opposed-phase image (**c**) and the T1w fs image (**d**), indicating fat within the tumor. After injection of 0.05 mmol/kg gadobenate dimeglumine, the lesion shows inhomogeneous but strong vascularization in the early arterial phase (**e**) with early wash-out in the later phase (**f**). In the fat suppressed image in the equilibrium phase (**g**) additional lesions (*arrows*) are visible that are hypointense compared with the normal renal parenchyma. Taken together these findings indicate multiple angiomyolipoma, which were proven after nephron sparing surgery of the largest lesion (performed because of the risk of bleeding)

**Fig. 4:**
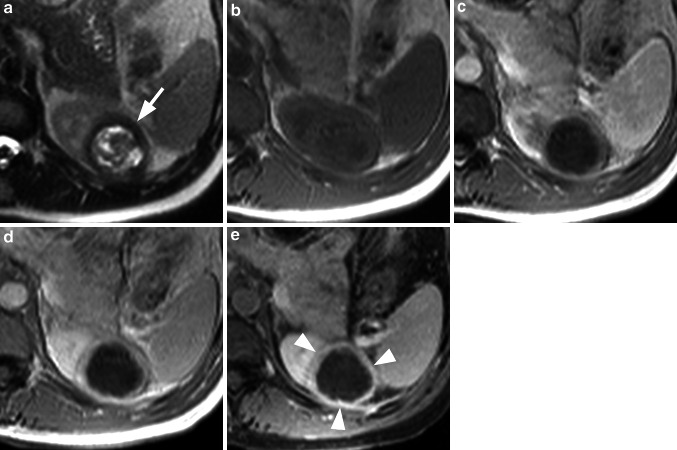
Renal abscess. The T2w HASTE sequence (**a**) shows an inhomogeneous cystic lesion (*arrow*) of the upper pole of the left kidney surrounded by a hypointense rim. The lesion is almost isointense to the renal cortex and medulla on the unenhanced T1w image (**b**). After injection of 0.05 mmol/kg gadobenate dimeglumine the rim surrounding the lesion shows, compared with the renal cortex, delayed but homogeneous contrast uptake (**c**, **d**), whereas the central areas remain hypointense without contrast enhancement. The T1w fs image during the equilibrium phase (**e**) shows persistent enhancement of the rim (*arrowheads*), indicating a wall of inflammatory tissue surrounding an abscess formation

**Fig. 5:**
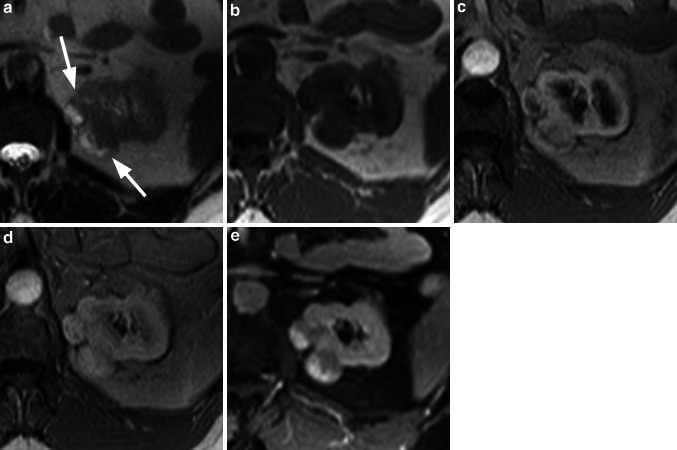
Multiple oncocytoma. The T2w HASTE sequence (**a**) shows two partially isointense and partially hyperintense lesions (*arrows*) at the upper pole of the left kidney. On the corresponding T1w image (**b**) the lesions are almost isointense to the renal cortex. After injection of 0.05 mmol/kg gadobenate dimeglumine, the lesions show an inhomogeneous hyervascularization in an early arterial phase (**c**) with homogenous contrast uptake in the later phase (**d**). On the T1w fs image (**e**) in the equilibrium phase the lesions show inhomogeneous contrast uptake. Diagnosis based on CE-MRI was multiple RCC; however, histology confirmed the lesions to be multiple oncocytoma

Based on CE-MRI findings alone, 39/62 (63 %) patients were diagnosed with malignant renal lesions. These 39 patients included 29 with RCC confirmed at histology (Figs. 1, 2), 7 patients with RCC confirmed at imaging follow-up, 2 patients with renal metastases confirmed at histology (in patients with primary lung cancer and breast cancer), and 1 patient with lymphoma confirmed at imaging follow-up. The 29 RCC confirmed at histology included 20 clear cell tumors, 6 papillary tumors, 1 mixed clear cell/papillary tumor, and 2 chromophobe tumors. These 39 patients were considered TP for renal cancer. A further 14/62 (22.6 %) patients were diagnosed with benign (*n* = 12) or no (*n* = 2) renal lesions. The 12 patients with benign lesions included 8 with complicated renal cysts confirmed at imaging follow-up (*n* = 7) or histology (*n* = 1), 3 patients with AML confirmed at imaging follow-up [including one 10-year-old boy with multiple bilateral AML (Fig. 3), one of which was resected due to its rapid growth and risk of hemorrhage], and 1 patient diagnosed with inflammatory tumor (Fig. 4) which was confirmed at imaging follow-up (pyelonephritis with renal abscess formation, which completely resolved under antibiotic therapy). These 14 patients were considered TN for malignant renal tumors. The remaining 9 patients included 8 patients that were diagnosed with malignant lesions (*n* = 7) or an unspecified lesion (*n* = 1) at CE-MRI which were confirmed as benign [oncocytoma in 5 patients (Fig. 5), atypical AML in 3 patients] at either histology (*n* = 6) or imaging follow-up (*n* = 2). One of these patients was diagnosed with both an RCC and AML which were confirmed as two AML at histology. These 8 patients were considered FP for malignant renal tumors. The final patient was considered to have an unspecific lesion at CE-MRI which was confirmed as an atypical RCC at histology. Based on its appearance at CE-MRI (solid, hypovascular, showing almost no contrast uptake, and without characteristic features of a benign lesion), the lesion was recommended for resection. Nevertheless, for the purposes of this study, since a malignant diagnosis was not made at CE-MRI, this patient was considered FN for malignant renal tumors.

The overall mean size of lesions was 27.1 ± 20.6 mm (range: 4–94 mm). The overall mean size of lesions diagnosed as malignant (27.6 ± 21.6 mm; range: 4–94 mm) was slightly larger than those diagnosed as benign (17.3 ± 16.2 mm; range: 4–67 mm).

Based on a total of 39 TP patients, 14 TN patients, 8 FP patients, and 1 FN patient, the overall sensitivity, specificity, accuracy, PPV, and NPV were determined to be 97.5 % (39/40), 63.6 % (14/22), 85.5 % (53/62), 83.0 % (39/47), and 93.3 % (14/15), respectively, for the diagnosis of renal cancer on CE-MRI with 0.05 mmol/kg gadobenate dimeglumine. No gender-based differences were apparent: the sensitivity, specificity, accuracy, PPV, and NPV for male patients [95.6 % (22/23), 71.4 % (10/14), 86.5 % (32/37), 84.6 % (22/26), and 90.9 % (10/11)] were similar to that of female patients [100 % (17/17), 50 % (4/8), 84 % (21/25), 80.9 % (17/21), and 100 % (4/4)].

**Fig. 6:**
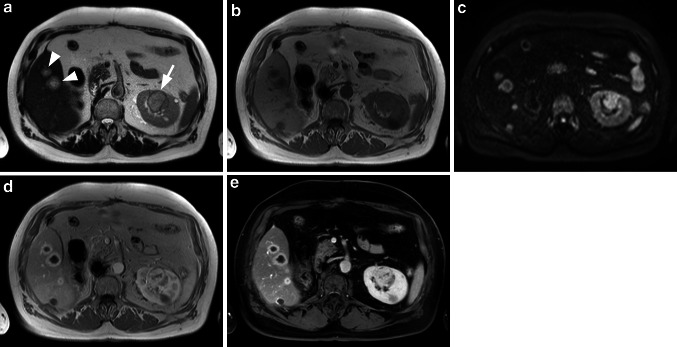
RCC with multiple liver metastases. The T2w HASTE sequence (**a**) reveals multiple liver metastases (*arrowheads*) from a RCC of the left kidney (*arrow*). On the corresponding T1w image (**b**) the liver lesions are hypointense to the liver parenchyma, whereas the RCC is isointense with the renal cortex. On the DWI image with a *b*-value of 800 (**c**) the liver metastases as well as the tumor in the left kidney are hyperintense indicating diffusion restriction. After injection of 0.05 mmol/kg gadobenate dimeglumine, the liver lesions as well as the RCC of the left kidney are hypervascular during the early arterial phase (**d**) with the RCC showing early contrast wash-out on the T1w fs image (**e**) during a later phase

Among the 37 patients with confirmed RCC (36 TP patients plus 1 FN patient), additional findings in the liver were noted in 6 patients [metastases in 3 patients (Fig. 6), hemangiomas in 3 patients].

### Blinded qualitative evaluation

The quality of kidney visualization was rated as excellent in 60 patients and as good in 2 patients. In no patient was the quality of visualization considered insufficient, poor, or moderate. Images were completely free of artifacts in 58 patients; in the remaining 4 patients, minimal artifacts were noted which did not compromise image interpretation or diagnosis. The necessary imaging information needed to make a diagnosis was considered complete in all 62 patients; the value of this diagnostic information was considered high in 61 patients and satisfactory in just one patient.

## Discussion

An early prospective assessment of the diagnostic performance of CE-MRI for the differentiation of patients with malignant renal lesions from patients with benign renal lesions determined values for sensitivity, specificity, PPV, and NPV of 93.8 % (15/16), 66.7 % (8/12), 78.9 % (15/19), and 88.8 % (8/9), respectively [9]. In that study, all oncocytomas were falsely classified as carcinomas (FP lesions) while three AMLs and three inflammatory lesions were correctly classified as benign (TN lesions) resulting in an overall per-patient-based accuracy of 82.1 % (23/28). In common with other studies focused on the CE-MRI evaluation of renal masses [13–18], the study utilized a conventional GBCA at a dose of 0.1 mmol/kg bodyweight [9]. Our retrospective study, performed as part of routine clinical practice in patients referred for abdominal/renal MRI because of unclear findings on multiphasic MDCT, utilized gadobenate dimeglumine at a dose of only 0.05 mmol/kg bodyweight. Our values for sensitivity, specificity, accuracy, PPV, and NPV of 97.5 % (39/40), 63.6 % (14/22), 85.5 % (53/62), 83.0 % (39/47), and 93.3 % (14/15), respectively, bear excellent comparison with those of the above study [9]. Although comparison with a full dose of another GBCA was not performed in our study, our results suggest that equivalent diagnostic performance might be achieved with just half the amount of gadolinium if gadobenate dimeglumine is the utilized GBCA. In this regard, a previous intra-individual crossover comparison of 0.05 mmol/kg gadobenate dimeglumine with 0.1 mmol/kg gadopentetate dimeglumine in patients undergoing CE-MRI of the liver revealed clear equivalence during the dynamic phase of contrast enhancement with significant superiority for gadobenate dimeglumine during the delayed hepatobiliary phase [21].

Clearly, these findings are potentially very important given the risk of nephrogenic systemic fibrosis (NSF) in patients with severe renal insufficiency given the possibility of compromised renal function in patients with renal tumors and the fact that new-onset post-operative chronic kidney disease (CKD) is a relatively frequent occurrence in patients undergoing curative surgery for renal cell carcinoma, particularly in patients aged 60 years or older and in patients with already decreased preoperative renal function [22–26]. In this regard, although symptoms of NSF typically manifest within approximately 3 months of GBCA administration, longer lead-times are not unknown [27, 28]. Notably, no unconfounded cases of NSF have yet been reported for gadobenate dimeglumine despite its regular use in patients at heightened risk of developing this disease [29–32]. As reported elsewhere for a variety of MR indications [21, 33–40], the possibility to use a reduced dose of gadobenate dimeglumine reflects the higher r1 relaxivity of this agent (6.2 vs. 3.9–4.6 L mmol^−1^ s^−1^ at 1.5 T according to recent data [20]) which derives from weak, transient interaction of the Gd-BOPTA contrast-effective molecule with serum albumin [41, 42] and a resulting slowing of the molecular tumbling rate which leads to greater shortening of the T1 relaxation time and thus greater signal enhancement at equivalent dose [43].

Unlike conventional GBCAs which are excreted almost exclusively via the kidneys, approximately 2–4 % of the injected dose of gadobenate dimeglumine is eliminated via the hepatobiliary pathway [44, 45]. Although this level of hepatobiliary excretion does not alter the pharmacokinetic profile of gadobenate dimeglumine relative to the profiles of conventional GBCAs [44, 45] or the characteristic dynamic enhancement patterns of frequently encountered tumors in the liver [46–51] and breast [52–55], it is sufficient to permit delayed hepatobiliary phase imaging of the liver for the improved detection and characterization of liver lesions [46–51]. On the one hand, the similar dynamic enhancement behavior of gadobenate dimeglumine relative to conventional GBCAs, even at half the dose [21], may explain the similar diagnostic performance of gadobenate dimeglumine for renal MRI not only in terms of the accurate detection of malignant lesions but also the accurate differentiation of malignant from benign lesions. In this regard, the FP lesions detected in our study (5 oncocytoma, 3 atypical AML i.e., AML without visible fat) are typical of the FP lesions detected elsewhere [9, 15, 17–19, 56, 57]. Both of these benign lesions share many radiologic features with certain types of RCC [12, 18, 58] making accurate and consistent differentiation challenging. Atypical AML without visible fat accounts for approximately 5 % of all AMLs [18, 56], while oncocytomas account for approximately 3–7 % of all adult renal neoplasms [59]. On the other hand, the additional possibility to acquire delayed hepatobiliary phase images of the liver during the same imaging session may be of value for patients diagnosed with aggressive and potentially metastatic RCC or with other incidental findings detected during the initial dynamic acquisition. In our study, 3/37 patients with RCC presented with additional liver metastases. Although in all cases these findings were noted on dynamic phase acquisitions of the liver as hypervascular lesions, reflecting the typical enhancement pattern of metastases from RCC, such patients may benefit from an additional hepatobiliary phase exam at no extra cost or inconvenience and with no additional GBCA administration, particularly given that as many as 30 % of patients with RCC present with metastases [60–62] and that metastatic RCC has a relatively poor prognosis (5-year survival rate approximately 20 % [63, 64]). Several studies have shown that hepatobiliary phase imaging with gadobenate dimeglumine improves the detection of hepatic metastases relative to that achievable with other techniques [65–67]. A further 3 patients with RCC in our study had liver hemangiomas detected incidentally during dynamic phase imaging. Characterization of these lesions and differentiation from liver metastases was correctly achieved in all cases reflecting the suitability of gadobenate dimeglumine for liver imaging. In this regard, gadobenate dimeglumine is specifically approved in Europe and elsewhere for MR imaging of the liver at a dose of 0.05 mmol/kg bodyweight [68]. As noted elsewhere [29, 31, 69], the partial hepatobiliary elimination of gadobenate dimeglumine combined with the administration of a reduced dose in at-risk patients may in part explain the absence of NSF in patients given this GBCA.

Our study has several limitations. First, it was a retrospective assessment of consecutive patients rather than a prospective evaluation. However, it should be noted that our findings for diagnostic performance were similar to those obtained elsewhere in a smaller prospective study [9]. Second, the evaluation of image quality was subjective rather than objective based on pre-defined quantitative or qualitative measures of enhancement. Finally, DWI was performed in only 29 patients in our study. Numerous studies have demonstrated the value of DWI for the improved characterization of detected renal neoplasms [15, 16, 57, 70–74]. One such study [15] showed that the addition of ADC information to CE-MRI findings led to the reclassification of 3 of 6 oncocytomas and 2 of 2 multilocular cystic nephromas that had originally been misdiagnosed as malignant on CE-MRI alone, thereby increasing specificity from 89 to 96 %. The use of gadobenate dimeglumine for DWI has previously been demonstrated in 83 patients with 85 renal masses [75]. In that study, a dose of 0.1 mmol/kg gadobenate dimeglumine enabled sensitive and specific differentiation of clear cell, papillary, and chromophobic RCC. Specifically, the sensitivity and specificity values for the differentiation of clear cell from non-clear cell RCC were high (95.9 and 94.4 %, respectively), which is particularly important given that patients with chromophobic or papillary RCC (i.e., not clear cell RCC) have a better prognosis than patients with clear cell RCC [76] and that these subtypes respond differently to molecularly targeted therapies.

In conclusion, our study confirms that gadobenate dimeglumine at a dose of 0.05 mmol/kg bodyweight is appropriate for MR imaging of the kidney, for the detection and differential diagnosis of renal neoplasms with high diagnostic performance.
